# *trans*-Dichlorido­bis­[tris(4-meth­oxy­phenyl)­phosphane]palladium(II) toluene solvate

**DOI:** 10.1107/S1600536810040912

**Published:** 2010-10-20

**Authors:** Alfred Muller, Reinout Meijboom

**Affiliations:** a Research Centre for Synthesis and Catalysis, Department of Chemistry, University of Johannesburg, PO Box 524 Auckland Park, Johannesburg, 2006, South Africa

## Abstract

In the title compound, *trans*-[PdCl_2_{P(4-MeOC_6_H_4_)_3_}_2_]·C_7_H_8_, the Pd(II) atom lies on a center of symmetry, resulting in a distorted *trans*-square planar geometry. The Pd—P and Pd—Cl bond lengths are 2.3409 (4) and 2.2981 (4) Å, respectively. An intra­molecular C—H⋯Cl hydrogen bond occurs. In the crystal, weak C—H⋯O inter­actions are observed between the aromatic rings of adjacent mol­ecules. The toluene solvate molecule is equally disordered over two sets of sites.

## Related literature

For a review on related compounds, see: Spessard & Miessler (1996 ▶). For related compounds, see: Meijboom & Omondi (2010 ▶). For the synthesis of the starting materials, see: Drew & Doyle (1990 ▶).
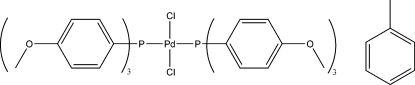


## Experimental

### 

#### Crystal data

[PdCl_2_(C_21_H_21_O_3_P)_2_]·C_7_H_8_
*M* *_r_* = 974.13
Triclinic, 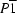

*a* = 7.8545 (4) Å
*b* = 12.1231 (7) Å
*c* = 12.4024 (8) Å
α = 85.666 (2)°
β = 78.762 (2)°
γ = 75.919 (2)°
*V* = 1123.03 (11) Å^3^
*Z* = 1
Mo *K*α radiation
μ = 0.65 mm^−1^
*T* = 100 K
0.27 × 0.20 × 0.08 mm

#### Data collection

Bruker X8 APEXII 4K Kappa CCD diffractometer
Absorption correction: multi-scan (*SADABS*; Bruker, 2004 ▶) *T*_min_ = 0.844, *T*_max_ = 0.950
19639 measured reflections
5573 independent reflections
5169 reflections with *I* > 2σ(*I*)
*R*_int_ = 0.037

#### Refinement

*R*[*F* ^2^ > 2σ(*F* ^2^)] = 0.029
*wR*(*F* ^2^) = 0.074
*S* = 1.06
5573 reflections
273 parameters
4 restraints
H-atom parameters constrained
Δρ_max_ = 1.28 e Å^−3^
Δρ_min_ = −0.67 e Å^−3^

### 

Data collection: *APEX2* (Bruker, 2005 ▶); cell refinement: *SAINT-Plus* (Bruker, 2004 ▶); data reduction: *SAINT-Plus* and *XPREP* (Bruker, 2004 ▶); program(s) used to solve structure: *SIR97* (Altomare *et al.*, 1999 ▶); program(s) used to refine structure: *SHELXL97* (Sheldrick, 2008 ▶); molecular graphics: *DIAMOND* (Brandenburg & Putz, 2005 ▶); software used to prepare material for publication: *WinGX* (Farrugia, 1999 ▶).

## Supplementary Material

Crystal structure: contains datablocks global, I. DOI: 10.1107/S1600536810040912/hg2723sup1.cif

Structure factors: contains datablocks I. DOI: 10.1107/S1600536810040912/hg2723Isup2.hkl

Additional supplementary materials:  crystallographic information; 3D view; checkCIF report

## Figures and Tables

**Table 1:** Hydrogen-bond geometry (Å, °)

*D*—H⋯*A*	*D*—H	H⋯*A*	*D*⋯*A*	*D*—H⋯*A*
C01—H01*B*⋯O2^i^	0.98	2.36	3.327 (7)	170
C3—H3*A*⋯O2^ii^	0.98	2.57	3.255 (3)	127
C36—H36⋯Cl	0.95	2.79	3.5402 (19)	136

Symmetry codes: (i) 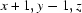; (ii) 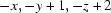.

**Fig. 1.**
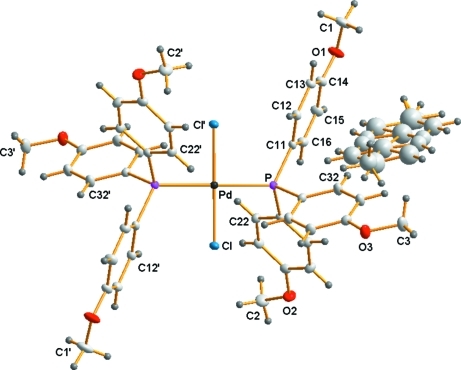
The structure (I), showing 50% probability displacement ellipsoids. For the C atoms, the first digit indicates ring number and the second digit indicates the position of the atom in the ring. Accented lettering indicate atoms generated by symmetry (1 - x,1 - y,1 - z).

## supplementary crystallographic information

### Comment

Transition metal complexes containing phosphine, arsine and stibine ligands are widely being investigated in various fields of organometallic chemistry (Spessard & Miessler, 1996). As part of a systematic investigation involving complexes with the general formula *trans*-[*MX*_2_(*L*)_2_] (*M* = Pt or Pd; *X* = halogen, Me, Ph; *L* = Group 15 donor ligand), crystals of the title compound, were obtained.

[PdCl_2_(*L*)_2_] (*L* = tertiary phosphine, arsine or stibine) complexes can conveniently be prepared by the substitution of 1,5-cyclooctadiene (COD) from [PdCl_2_(COD)]. The title compound, *trans*-[PdCl_2_{P(4-MeOC_6_H_4_)_3_}_2_], crystallizes in the triclinic spacegroup *P*1, with the Pd atom on a center of symmetry and each pair of equivalent ligands in a mutually *trans* orientation. The geometry is, therefore, slightly distorted square planar and the Pd atom is not elevated out of the coordinating atom plane. All angles in the coordination polyhedron are close to the ideal value of 90°, with P—Pd—Cl = 88.422 (15) and P—Pd—Cl^i^ = 91.578 (15)°. As required by the crystallographic symmetry, the P—Pd—P^i^ and Cl—Pd—Cl^i^ angles are 180°. Some weak intermolecular interactions were observed and are reported in Table 1.

The title compound compares well with other closely related Pd^II^ complexes from the literature containing two chloro and two tertiary phosphine ligands in a *trans* geometry. The title compound, having a Pd—Cl bond length of 2.2981 (4) Å and a Pd—P bond length of 2.3409 (4) Å, fits well into the typical range for complexes of this kind. Notably the title compound crystallized as a solvated complex; these type of Pd^II^ complexes have a tendency to crystallize as solvates (Meijboom & Omondi, 2010). The solvate molecule, toluene, is found 50:50 disordered molecule.

### Experimental

Dichloro(1,5-cyclooctadiene)palladium(II), [PdCl_2_(COD)], was prepared according to the literature procedure of Drew & Doyle (1990). A solution of tris(4-methoxyphenyl)phosphine (0.2 mmol) in dichloromethane (2.0 cm^3^) was added to a solution of [PdCl_2_(COD)] (0.1 mmol) in dichloromethane (3.0 cm^3^). Slow evaporation of the solvent gave the parent palladium compound. Recrystallization from tolunene/hexane afforded crystals of the title compound.

### Refinement

The aromatic and methyl H atoms were placed in geometrically idealized positions (C—H = 0.95–0.98) and constrained to ride on their parent atoms with *U*_iso_(H) = 1.2*U*_eq_(C) for aromatic and *U*_iso_(H) = 1.5*U*_eq_(C) for methyl H atoms respectively. Methyl torsion angles were refined from electron density

### Crystal data

**Table:**

[PdCl_2_(C_21_H_21_O_3_P)_2_]·C_7_H_8_	*Z* = 1
*M**_r_* = 974.13	*F*(000) = 502
Triclinic, *P*1	*D*_x_ = 1.44 Mg m^−^^3^
Hall symbol: -P 1	Mo *K*α radiation, λ = 0.71073 Å
*a* = 7.8545 (4) Å	Cell parameters from 5142 reflections
*b* = 12.1231 (7) Å	θ = 2.4–28.3°
*c* = 12.4024 (8) Å	µ = 0.65 mm^−^^1^
α = 85.666 (2)°	*T* = 100 K
β = 78.762 (2)°	Plate, yellow
γ = 75.919 (2)°	0.27 × 0.2 × 0.08 mm
*V* = 1123.03 (11) Å^3^	

### Data collection

**Table:**

Bruker X8 APEXII 4K Kappa CCD diffractometer	5573 independent reflections
Radiation source: fine-focus sealed tube	5169 reflections with *I* > 2σ(*I*)
graphite	*R*_int_ = 0.037
Detector resolution: 8.4 pixels mm^-1^	θ_max_ = 28.3°, θ_min_ = 2.4°
φ and ω scans	*h* = −10→10
Absorption correction: multi-scan (*SADABS*; Bruker, 2004)	*k* = −16→16
*T*_min_ = 0.844, *T*_max_ = 0.950	*l* = −16→16
19639 measured reflections	

### Refinement

**Table:**

Refinement on *F*^2^	Primary atom site location: structure-invariant direct methods
Least-squares matrix: full	Secondary atom site location: difference Fourier map
*R*[*F*^2^ > 2σ(*F*^2^)] = 0.029	Hydrogen site location: inferred from neighbouring sites
*wR*(*F*^2^) = 0.074	H-atom parameters constrained
*S* = 1.06	*w* = 1/[σ^2^(*F*_o_^2^) + (0.0261*P*)^2^ + 1.197*P*] where *P* = (*F*_o_^2^ + 2*F*_c_^2^)/3
5573 reflections	(Δ/σ)_max_ < 0.001
273 parameters	Δρ_max_ = 1.27 e Å^−^^3^
4 restraints	Δρ_min_ = −0.67 e Å^−^^3^

### Special details

**Table:**

Experimental. The intensity data was collected on a Bruker X8 Apex II 4 K Kappa CCD diffractometer using an exposure time of 6 s/frame. A total of 1637 frames were collected with a frame width of 0.5° covering up to θ = 28.31° with 99.8% completeness accomplished.
Geometry. All e.s.d.'s (except the e.s.d. in the dihedral angle between two l.s. planes) are estimated using the full covariance matrix. The cell e.s.d.'s are taken into account individually in the estimation of e.s.d.'s in distances, angles and torsion angles; correlations between e.s.d.'s in cell parameters are only used when they are defined by crystal symmetry. An approximate (isotropic) treatment of cell e.s.d.'s is used for estimating e.s.d.'s involving l.s. planes.
Refinement. Refinement of *F*^2^ against ALL reflections. The weighted *R*-factor *wR* and goodness of fit *S* are based on *F*^2^, conventional *R*-factors *R* are based on *F*, with *F* set to zero for negative *F*^2^. The threshold expression of *F*^2^ > σ(*F*^2^) is used only for calculating *R*-factors(gt) *etc*. and is not relevant to the choice of reflections for refinement. *R*-factors based on *F*^2^ are statistically about twice as large as those based on *F*, and *R*- factors based on ALL data will be even larger.

### Fractional atomic coordinates and isotropic or equivalent isotropic displacement parameters (Å^2^)

**Table:**

	*x*	*y*	*z*	*U*_iso_*/*U*_eq_	Occ. (<1)
Pd	0.5	0.5	0.5	0.01073 (6)	
P	0.32288 (6)	0.43533 (4)	0.65434 (4)	0.01130 (9)	
Cl	0.44125 (6)	0.67186 (4)	0.58287 (4)	0.01752 (10)	
C11	0.2359 (2)	0.31163 (15)	0.64258 (14)	0.0129 (3)	
C12	0.3519 (2)	0.20484 (15)	0.62301 (15)	0.0150 (3)	
H12	0.4766	0.1972	0.6168	0.018*	
C13	0.2882 (2)	0.10942 (15)	0.61244 (15)	0.0162 (3)	
H13	0.3689	0.0375	0.599	0.019*	
C14	0.1045 (3)	0.11999 (16)	0.62176 (16)	0.0179 (4)	
C15	−0.0126 (3)	0.22615 (16)	0.64053 (17)	0.0198 (4)	
H15	−0.1373	0.2339	0.6462	0.024*	
C16	0.0528 (2)	0.32029 (15)	0.65097 (15)	0.0159 (3)	
H16	−0.0282	0.3922	0.6641	0.019*	
C21	0.1254 (2)	0.54510 (14)	0.70174 (14)	0.0124 (3)	
C22	0.0282 (2)	0.60449 (16)	0.62413 (15)	0.0161 (3)	
H22	0.0638	0.5836	0.5493	0.019*	
C23	−0.1195 (2)	0.69346 (15)	0.65361 (15)	0.0163 (3)	
H23	−0.187	0.7315	0.6002	0.02*	
C24	−0.1672 (2)	0.72600 (15)	0.76289 (15)	0.0159 (3)	
C25	−0.0732 (3)	0.66642 (16)	0.84167 (15)	0.0180 (4)	
H25	−0.1083	0.6878	0.9163	0.022*	
C26	0.0716 (2)	0.57583 (15)	0.81157 (15)	0.0151 (3)	
H26	0.1343	0.5347	0.8658	0.018*	
C31	0.4441 (2)	0.39989 (15)	0.76733 (14)	0.0131 (3)	
C32	0.3972 (3)	0.32619 (17)	0.85375 (15)	0.0186 (4)	
H32	0.3015	0.2914	0.8524	0.022*	
C33	0.4873 (3)	0.30242 (17)	0.94193 (16)	0.0194 (4)	
H33	0.4539	0.2516	0.9999	0.023*	
C34	0.6271 (2)	0.35369 (16)	0.94457 (15)	0.0169 (4)	
C35	0.6740 (3)	0.42844 (16)	0.85931 (15)	0.0180 (4)	
H35	0.7683	0.4642	0.8614	0.022*	
C36	0.5843 (2)	0.45100 (16)	0.77175 (15)	0.0161 (3)	
H36	0.6181	0.5018	0.7139	0.019*	
C1	0.1436 (3)	−0.07965 (17)	0.6040 (2)	0.0291 (5)	
H1A	0.2311	−0.082	0.5355	0.044*	
H1B	0.0727	−0.1355	0.602	0.044*	
H1C	0.206	−0.0977	0.6665	0.044*	
O1	0.0278 (2)	0.03200 (12)	0.61544 (14)	0.0259 (3)	
C2	−0.3886 (3)	0.88867 (18)	0.72033 (18)	0.0257 (4)	
H2A	−0.3003	0.9208	0.6689	0.039*	
H2B	−0.4815	0.9505	0.7574	0.039*	
H2C	−0.4428	0.8442	0.6799	0.039*	
O2	−0.30260 (18)	0.81656 (12)	0.80035 (12)	0.0217 (3)	
C3	0.6746 (3)	0.2635 (2)	1.11707 (17)	0.0274 (5)	
H3A	0.5497	0.2941	1.1507	0.041*	
H3B	0.7509	0.2604	1.1716	0.041*	
H3C	0.6883	0.1867	1.0916	0.041*	
O3	0.7257 (2)	0.33564 (13)	1.02543 (11)	0.0231 (3)	
C01	0.7355 (9)	−0.0476 (6)	1.0145 (5)	0.0481 (14)*	0.5
H01A	0.7192	−0.1018	1.0762	0.072*	0.5
H01B	0.734	−0.0824	0.946	0.072*	0.5
H01C	0.6386	0.0211	1.0261	0.072*	0.5
C02	0.9034 (10)	−0.0180 (6)	1.0076 (6)	0.0624 (18)*	0.5
C03	0.9981 (10)	−0.0566 (5)	1.0829 (5)	0.0409 (14)*	0.5
H03	0.9624	−0.1095	1.1379	0.049*	0.5
C04	1.1548 (11)	−0.0191 (6)	1.0822 (6)	0.0619 (18)*	0.5
H04	1.2331	−0.0426	1.1334	0.074*	0.5
C05	1.1757 (14)	0.0568 (8)	0.9956 (8)	0.074 (2)*	0.5
H05	1.2674	0.0949	0.9974	0.088*	0.5
C06	1.0987 (9)	0.0869 (6)	0.9139 (6)	0.0521 (15)*	0.5
H06	1.1435	0.1327	0.8548	0.063*	0.5
C07	0.9368 (10)	0.0471 (5)	0.9159 (5)	0.0410 (14)*	0.5
H07	0.865	0.0644	0.8607	0.049*	0.5

### Atomic displacement parameters (Å^2^)

**Table:**

	*U*^11^	*U*^22^	*U*^33^	*U*^12^	*U*^13^	*U*^23^
Pd	0.01295 (9)	0.00955 (9)	0.00981 (9)	−0.00301 (6)	−0.00176 (6)	−0.00060 (6)
P	0.0125 (2)	0.0109 (2)	0.0107 (2)	−0.00299 (16)	−0.00240 (15)	0.00014 (15)
Cl	0.0238 (2)	0.0120 (2)	0.0163 (2)	−0.00615 (16)	0.00094 (16)	−0.00323 (15)
C11	0.0160 (8)	0.0127 (8)	0.0108 (7)	−0.0056 (6)	−0.0026 (6)	0.0017 (6)
C12	0.0149 (8)	0.0147 (8)	0.0157 (8)	−0.0044 (7)	−0.0034 (6)	0.0012 (6)
C13	0.0194 (9)	0.0117 (8)	0.0172 (8)	−0.0027 (7)	−0.0046 (7)	0.0009 (6)
C14	0.0232 (9)	0.0140 (8)	0.0196 (9)	−0.0072 (7)	−0.0086 (7)	0.0018 (7)
C15	0.0156 (9)	0.0180 (9)	0.0278 (10)	−0.0054 (7)	−0.0070 (7)	−0.0003 (7)
C16	0.0155 (8)	0.0136 (8)	0.0186 (9)	−0.0020 (7)	−0.0046 (7)	−0.0010 (7)
C21	0.0119 (8)	0.0110 (8)	0.0148 (8)	−0.0034 (6)	−0.0029 (6)	0.0002 (6)
C22	0.0189 (9)	0.0165 (9)	0.0132 (8)	−0.0034 (7)	−0.0042 (7)	−0.0008 (6)
C23	0.0173 (9)	0.0148 (8)	0.0172 (8)	−0.0023 (7)	−0.0073 (7)	0.0017 (7)
C24	0.0126 (8)	0.0145 (8)	0.0208 (9)	−0.0033 (6)	−0.0028 (7)	−0.0022 (7)
C25	0.0173 (9)	0.0212 (9)	0.0147 (8)	−0.0021 (7)	−0.0025 (7)	−0.0039 (7)
C26	0.0153 (8)	0.0161 (8)	0.0141 (8)	−0.0023 (7)	−0.0045 (6)	−0.0003 (6)
C31	0.0134 (8)	0.0138 (8)	0.0115 (8)	−0.0016 (6)	−0.0033 (6)	−0.0005 (6)
C32	0.0196 (9)	0.0223 (9)	0.0173 (9)	−0.0108 (7)	−0.0056 (7)	0.0043 (7)
C33	0.0233 (10)	0.0206 (9)	0.0174 (9)	−0.0104 (8)	−0.0067 (7)	0.0049 (7)
C34	0.0180 (9)	0.0192 (9)	0.0147 (8)	−0.0041 (7)	−0.0055 (7)	−0.0014 (7)
C35	0.0172 (9)	0.0210 (9)	0.0185 (9)	−0.0088 (7)	−0.0054 (7)	0.0012 (7)
C36	0.0166 (8)	0.0170 (9)	0.0150 (8)	−0.0052 (7)	−0.0032 (7)	0.0024 (7)
C1	0.0310 (11)	0.0126 (9)	0.0482 (14)	−0.0063 (8)	−0.0169 (10)	0.0006 (9)
O1	0.0244 (7)	0.0132 (7)	0.0448 (9)	−0.0067 (6)	−0.0144 (7)	−0.0006 (6)
C2	0.0239 (10)	0.0197 (10)	0.0302 (11)	0.0043 (8)	−0.0088 (8)	−0.0007 (8)
O2	0.0191 (7)	0.0194 (7)	0.0228 (7)	0.0045 (5)	−0.0052 (5)	−0.0033 (5)
C3	0.0359 (12)	0.0337 (12)	0.0201 (10)	−0.0178 (10)	−0.0149 (9)	0.0094 (8)
O3	0.0266 (7)	0.0310 (8)	0.0178 (7)	−0.0141 (6)	−0.0122 (6)	0.0064 (6)

### Geometric parameters (Å, °)

**Table:**

Pd—Cl	2.2981 (4)	C33—H33	0.95
Pd—Cl^i^	2.2981 (4)	C34—O3	1.356 (2)
Pd—P^i^	2.3409 (4)	C34—C35	1.393 (3)
Pd—P	2.3409 (4)	C35—C36	1.382 (3)
P—C21	1.8112 (17)	C35—H35	0.95
P—C31	1.8124 (18)	C36—H36	0.95
P—C11	1.8185 (18)	C1—O1	1.435 (2)
C11—C12	1.397 (2)	C1—H1A	0.98
C11—C16	1.400 (2)	C1—H1B	0.98
C12—C13	1.391 (2)	C1—H1C	0.98
C12—H12	0.95	C2—O2	1.431 (2)
C13—C14	1.399 (3)	C2—H2A	0.98
C13—H13	0.95	C2—H2B	0.98
C14—O1	1.360 (2)	C2—H2C	0.98
C14—C15	1.394 (3)	C3—O3	1.435 (2)
C15—C16	1.384 (3)	C3—H3A	0.98
C15—H15	0.95	C3—H3B	0.98
C16—H16	0.95	C3—H3C	0.98
C21—C22	1.394 (2)	C01—C02	1.434 (10)
C21—C26	1.396 (2)	C01—H01A	0.98
C22—C23	1.389 (3)	C01—H01B	0.98
C22—H22	0.95	C01—H01C	0.98
C23—C24	1.394 (3)	C02—C03	1.296 (10)
C23—H23	0.95	C02—C07	1.353 (10)
C24—O2	1.367 (2)	C03—C04	1.410 (10)
C24—C25	1.391 (3)	C03—H03	0.95
C25—C26	1.389 (2)	C04—C05	1.372 (12)
C25—H25	0.95	C04—H04	0.95
C26—H26	0.95	C05—C06	1.265 (11)
C31—C32	1.394 (2)	C05—H05	0.95
C31—C36	1.400 (3)	C06—C07	1.462 (9)
C32—C33	1.391 (3)	C06—H06	0.95
C32—H32	0.95	C07—H07	0.95
C33—C34	1.393 (3)		
			
Cl—Pd—Cl^i^	180	C34—C33—H33	120.3
Cl—Pd—P^i^	91.578 (15)	O3—C34—C35	115.85 (17)
Cl^i^—Pd—P^i^	88.422 (15)	O3—C34—C33	124.53 (17)
Cl—Pd—P	88.422 (15)	C35—C34—C33	119.62 (17)
Cl^i^—Pd—P	91.578 (15)	C36—C35—C34	120.49 (17)
P^i^—Pd—P	180.00 (2)	C36—C35—H35	119.8
C21—P—C31	106.76 (8)	C34—C35—H35	119.8
C21—P—C11	103.93 (8)	C35—C36—C31	120.74 (17)
C31—P—C11	105.02 (8)	C35—C36—H36	119.6
C21—P—Pd	110.78 (6)	C31—C36—H36	119.6
C31—P—Pd	110.54 (6)	O1—C1—H1A	109.5
C11—P—Pd	118.96 (6)	O1—C1—H1B	109.5
C12—C11—C16	118.07 (16)	H1A—C1—H1B	109.5
C12—C11—P	120.39 (14)	O1—C1—H1C	109.5
C16—C11—P	121.53 (13)	H1A—C1—H1C	109.5
C13—C12—C11	121.28 (17)	H1B—C1—H1C	109.5
C13—C12—H12	119.4	C14—O1—C1	117.21 (16)
C11—C12—H12	119.4	O2—C2—H2A	109.5
C12—C13—C14	119.65 (17)	O2—C2—H2B	109.5
C12—C13—H13	120.2	H2A—C2—H2B	109.5
C14—C13—H13	120.2	O2—C2—H2C	109.5
O1—C14—C15	115.85 (17)	H2A—C2—H2C	109.5
O1—C14—C13	124.46 (17)	H2B—C2—H2C	109.5
C15—C14—C13	119.68 (17)	C24—O2—C2	117.50 (15)
C16—C15—C14	120.00 (18)	O3—C3—H3A	109.5
C16—C15—H15	120	O3—C3—H3B	109.5
C14—C15—H15	120	H3A—C3—H3B	109.5
C15—C16—C11	121.33 (17)	O3—C3—H3C	109.5
C15—C16—H16	119.3	H3A—C3—H3C	109.5
C11—C16—H16	119.3	H3B—C3—H3C	109.5
C22—C21—C26	118.89 (16)	C34—O3—C3	117.05 (15)
C22—C21—P	118.30 (13)	C02—C01—H01A	109.5
C26—C21—P	122.75 (14)	C02—C01—H01B	109.5
C23—C22—C21	121.52 (17)	H01A—C01—H01B	109.5
C23—C22—H22	119.2	C02—C01—H01C	109.5
C21—C22—H22	119.2	H01A—C01—H01C	109.5
C22—C23—C24	118.83 (17)	H01B—C01—H01C	109.5
C22—C23—H23	120.6	C03—C02—C07	130.7 (7)
C24—C23—H23	120.6	C03—C02—C01	119.1 (7)
O2—C24—C25	115.53 (16)	C07—C02—C01	110.2 (7)
O2—C24—C23	124.16 (17)	C02—C03—C04	119.2 (7)
C25—C24—C23	120.30 (17)	C02—C03—H03	120.4
C26—C25—C24	120.24 (17)	C04—C03—H03	120.4
C26—C25—H25	119.9	C05—C04—C03	108.9 (8)
C24—C25—H25	119.9	C05—C04—H04	125.5
C25—C26—C21	120.14 (17)	C03—C04—H04	125.5
C25—C26—H26	119.9	C06—C05—C04	133.6 (10)
C21—C26—H26	119.9	C06—C05—H05	113.2
C32—C31—C36	118.22 (16)	C04—C05—H05	113.2
C32—C31—P	121.90 (14)	C05—C06—C07	116.0 (8)
C36—C31—P	119.82 (13)	C05—C06—H06	122
C33—C32—C31	121.47 (17)	C07—C06—H06	122
C33—C32—H32	119.3	C02—C07—C06	110.4 (7)
C31—C32—H32	119.3	C02—C07—H07	124.8
C32—C33—C34	119.46 (17)	C06—C07—H07	124.8
C32—C33—H33	120.3		
			
Cl—Pd—P—C21	40.71 (6)	C23—C24—C25—C26	1.6 (3)
Cl^i^—Pd—P—C21	−139.29 (6)	C24—C25—C26—C21	0.9 (3)
Cl—Pd—P—C31	−77.43 (6)	C22—C21—C26—C25	−1.9 (3)
Cl^i^—Pd—P—C31	102.57 (6)	P—C21—C26—C25	175.13 (14)
Cl—Pd—P—C11	160.99 (7)	C21—P—C31—C32	81.11 (16)
Cl^i^—Pd—P—C11	−19.01 (7)	C11—P—C31—C32	−28.83 (17)
C21—P—C11—C12	−170.72 (14)	Pd—P—C31—C32	−158.32 (14)
C31—P—C11—C12	−58.74 (16)	C21—P—C31—C36	−96.04 (15)
Pd—P—C11—C12	65.57 (16)	C11—P—C31—C36	154.02 (14)
C21—P—C11—C16	10.31 (17)	Pd—P—C31—C36	24.53 (16)
C31—P—C11—C16	122.28 (15)	C36—C31—C32—C33	−0.7 (3)
Pd—P—C11—C16	−113.40 (14)	P—C31—C32—C33	−177.86 (15)
C16—C11—C12—C13	−0.2 (3)	C31—C32—C33—C34	0.4 (3)
P—C11—C12—C13	−179.23 (14)	C32—C33—C34—O3	−179.07 (18)
C11—C12—C13—C14	−0.1 (3)	C32—C33—C34—C35	0.4 (3)
C12—C13—C14—O1	−178.36 (18)	O3—C34—C35—C36	178.74 (17)
C12—C13—C14—C15	0.6 (3)	C33—C34—C35—C36	−0.7 (3)
O1—C14—C15—C16	178.38 (18)	C34—C35—C36—C31	0.4 (3)
C13—C14—C15—C16	−0.6 (3)	C32—C31—C36—C35	0.3 (3)
C14—C15—C16—C11	0.3 (3)	P—C31—C36—C35	177.54 (14)
C12—C11—C16—C15	0.1 (3)	C15—C14—O1—C1	−175.70 (19)
P—C11—C16—C15	179.15 (15)	C13—C14—O1—C1	3.3 (3)
C31—P—C21—C22	166.77 (14)	C25—C24—O2—C2	172.50 (17)
C11—P—C21—C22	−82.53 (15)	C23—C24—O2—C2	−6.5 (3)
Pd—P—C21—C22	46.36 (15)	C35—C34—O3—C3	176.95 (18)
C31—P—C21—C26	−10.31 (17)	C33—C34—O3—C3	−3.6 (3)
C11—P—C21—C26	100.39 (16)	C07—C02—C03—C04	7.9 (11)
Pd—P—C21—C26	−130.73 (14)	C01—C02—C03—C04	−174.1 (6)
C26—C21—C22—C23	0.4 (3)	C02—C03—C04—C05	0.4 (9)
P—C21—C22—C23	−176.82 (14)	C03—C04—C05—C06	−9.8 (14)
C21—C22—C23—C24	2.2 (3)	C04—C05—C06—C07	10.3 (14)
C22—C23—C24—O2	175.75 (17)	C03—C02—C07—C06	−7.4 (10)
C22—C23—C24—C25	−3.2 (3)	C01—C02—C07—C06	174.4 (5)
O2—C24—C25—C26	−177.36 (17)	C05—C06—C07—C02	−1.1 (9)

Symmetry codes: (i) −*x*+1, −*y*+1, −*z*+1.

### Hydrogen-bond geometry (Å, °)

**Table:**

*D*—H···*A*	*D*—H	H···*A*	*D*···*A*	*D*—H···*A*
C01—H01B···O2^ii^	0.98	2.36	3.327 (7)	170
C3—H3A···O2^iii^	0.98	2.57	3.255 (3)	127
C36—H36···Cl	0.95	2.79	3.5402 (19)	136

Symmetry codes: (ii) *x*+1, *y*−1, *z*; (iii) −*x*, −*y*+1, −*z*+2.
